# Characterization of dental pulp stem/stromal cells of Huntington monkey tooth germs

**DOI:** 10.1186/1471-2121-12-39

**Published:** 2011-09-12

**Authors:** Brooke R Snyder, Pei-Hsun Cheng, Jinjing Yang, Shang-Hsun Yang, Anderson HC Huang, Anthony WS Chan

**Affiliations:** 1 Yerkes National Primate Research Center, 954 Gatewood Rd., N.E. Atlanta, GA 30329, USA; 2 Department of Human Genetics, Emory University School of Medicine, 615 Michael St. Whitehead Building, Atlanta, GA 30322, USA; 3 Department of Oral Pathology, School of Dentistry, Kaohsiung Medical University, 100 Shih-Chuan 1st Road, Kaohsiung, Taiwan, Republic of China

**Keywords:** Adult stem cells, animal model, DPSCs, Huntington's disease, transgenic HD monkeys, cell therapy

## Abstract

**Background:**

Dental pulp stem/stromal cells (DPSCs) are categorized as adult stem cells (ASCs) that retain multipotent differentiation capabilities. DPSCs can be isolated from individuals at any age and are considered to be true personal stem cells, making DPSCs one of the potential options for stem cell therapy. However, the properties of DPSCs from individuals with an inherited genetic disorder, such as Huntington's disease (HD), have not been fully investigated.

**Results:**

To examine if mutant huntingtin (htt) protein impacts DPSC properties, we have established DPSCs from tooth germ of transgenic monkeys that expressed both mutant *htt *and green fluorescent protein (*GFP*) genes (rHD/G-DPSCs), and from a monkey that expressed only the *GFP *gene (rG-DPSCs), which served as a control. Although mutant htt and oligomeric htt aggregates were overtly present in rHD/G-DPSCs, all rHD/G-DPSCs and rG-DPSCs shared similar characteristics, including self-renewal, multipotent differentiation capabilities, expression of stemness and differentiation markers, and cell surface antigen profile.

**Conclusions:**

Our results suggest that DPSCs from Huntington monkeys retain ASC properties. Thus DPSCs derived from individuals with genetic disorders such as HD could be a potential source of personal stem cells for therapeutic purposes.

## Background

DPSCs are ASCs and were first described in 2000 [1]. DPSCs share similar characteristics with bone marrow derived mesenchymal stem/stromal cells (BMSCs) [1-4]. DPSCs have been differentiated into neuronal cells under the guidance of neurogenic factors [5-10]. Additionally, DPSCs may also be therapeutic by providing trophic support [4,11] or recruiting endogenous cells for repair [4,12].

Most studies involving ASCs are derived from healthy donors, and studies involving DPSCs have, in general, been limited. Due to the advantages of using one's own cells for therapy [13,14], plus their potential use in the central nervous system (CNS) [4-9,15,16], we are interested in characterizing DPSCs derived from individuals with HD and determined if HD-DPSCs retain comparable stem cell properties to those derived from a healthy individual.

HD is a dominant genetic disorder caused by a mutation resulting in the expansion of polyglutamine (CAG) repeats in exon 1 of the IT15 gene encoding for htt. CAG repeat lengths over 39 results in pathological HD. A negative correlation has been shown between repeat length and age of onset [17] and lifespan [18]. The hallmark of HD is neurodegeneration, predominantly in the striatum and cortex [19]. Many HD patients do suffer from tooth decay; however this decay is due to medication and lack of motor skills to perform teeth maintenance procedures [20,21]. Here we have used transgenic HD monkeys [22] as a model for investigating the effect of mutant htt on the properties of DPSCs.

## Results

### Establishment of DPSCs from HD monkeys

**Figure 1:**
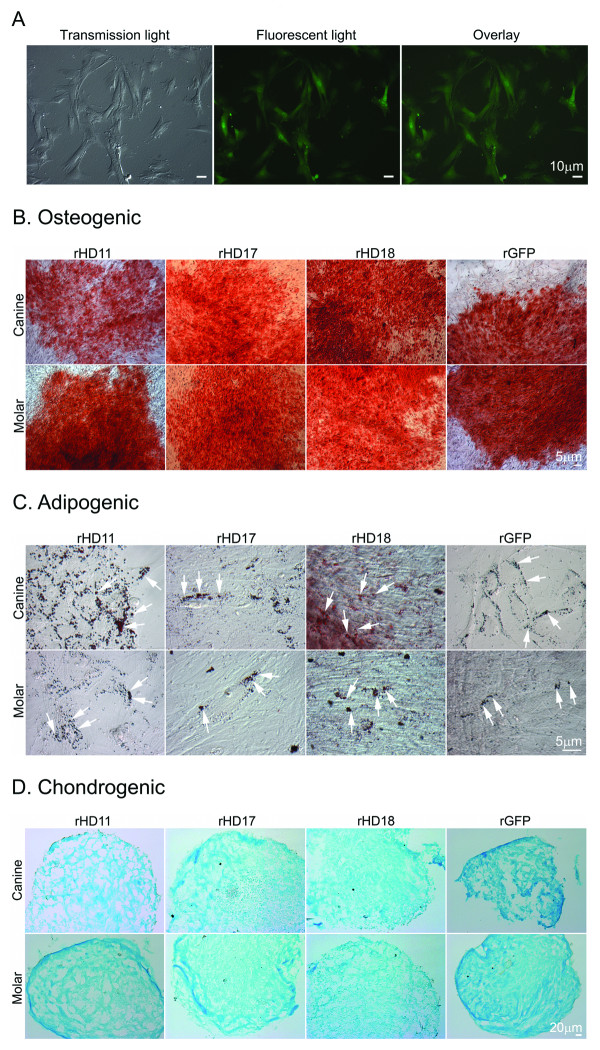
**Isolation, culture and differentiation of rHD/G-DPSCs**. (A) Spindle and fibroblast-like cells were observed in rHD/G-DPSC culture (Left), rHD/G-DPSCs express GFP (center) and overlay image (right). (B) Osteogenic differentiation, (C) Adipogenic differentiation, and (D) Chondrogenic differentiation of rHD/G-DPSCs and rG-DPSCs. Osteogenic and adipogenic differentiation were demonstrated by three weeks induction followed by Alizarin Red S and Oil-Red O staining, respectively. Chondrogenic differentiation was demonstrated by four weeks induction followed by Alcian blue staining of cryosection.

We have isolated and established DPSCs from the teeth buds of three double-transgenic HD/G monkeys (rHD11, rHD17 and rHD18) and one transgenic GFP monkey (rGFP). rHD17 and rHD18 died shortly after birth at full term. Both rHD17 and rHD18 demonstrated HD phenotypes including severe dystonia and chorea [22]. rHD11 and rGFP were miscarried monkeys, born at four months of gestation. rHD/G-DPSCs have distinctive morphology including long and spindle shaped (Figure 1A) which were similar to published reports of rBMSCs [23], rhesus monkey DPSCs (rDPSCs) [4], Chimpanzee DPSCs (ChDPSCs) [2], hBMSCs and hDPSCs [3,24,25]. There were no overt differences in cellular characteristics of DPSCs derived from different individuals. All cell lines expressed GFP (Figure 1A) and were capable of self-renewal.

### Expression of stem cell specific transcription factors in rHD/G-DPSCs

**Figure 2:**
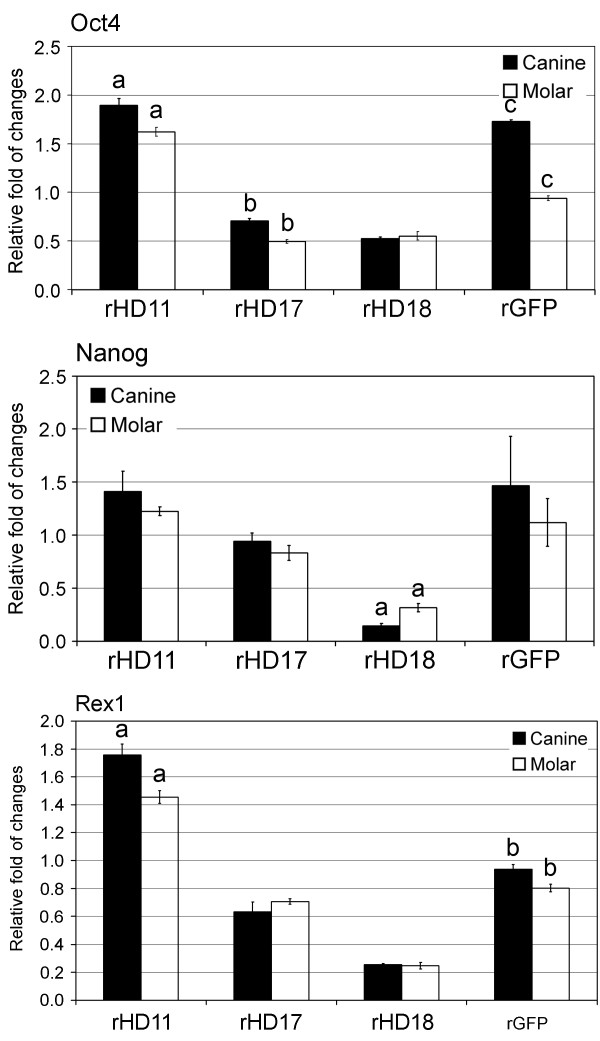
**Expression of stemness factors**. Quantitative analysis of the transcripts of stemness factors (Oct4, Nanog and Rex1) in canine and molar of rHD/G-DPSCs and rG-DPSCs was determined by qRT-PCR. Column shares the same alphabet are significantly different (P < 0.05). Three replicas were performed.

To further evaluate stem cell properties of rHD/G-DPSCs with various degree of mutant htt, quantitative measurement of stemness factors (Oct-4, Nanog and Rex-1; Figure 2) was performed by Q-PCR. There was no different in the expression pattern of stemness factors between canine and molar derived DPSCs. However, variations in expression levels were observed among individuals (Figure 2). rHD17 and rHD18 have lower expression compared to rHD11 and rGFP.

### Differentiation competence of rHD/GFP-DPSCs

One of the defining characteristics of ASCs is multipotent differentiation capability. All rHD/G-DPSCs were capable of differentiating into osteogenic (Figure 1B), adipogenic (Figure 1C) and chondrogenic lineages (Figure 1D), which are trademark events of ASCs [24,26] and DPSCs [3,4]. The multipotent differentiation capability of rHD/G-DPSCs and r/G-DPSCs were similar to published reports of rhesus macaques [4,23], chimpanzee [2] and humans [3,24] ASCs. Similar to previous studies, the fat droplets in DPSCs were relatively smaller and less intense than that of BMSCs [2,4].

**Figure 3:**
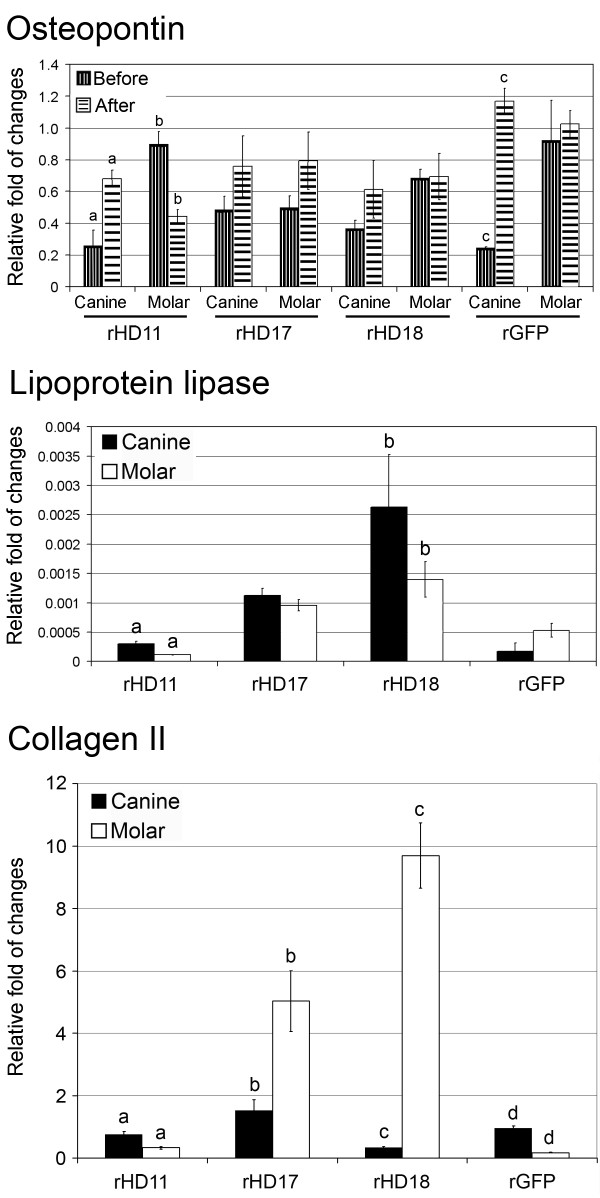
**Expression of differentiation markers**. Quantitative analysis of the transcripts of differentiation markers (Osteopontin, Lipoprotein lipase and Collagen II) in canine and molar of rHD/G-DPSCs and rG-DPSCs was determined by qRT-PCR. No detectable expression of lipoprotein lipase and collagen II was observed before induction of differentiation. Column shares the same alphabet are significantly different (P < 0.05). Three replicas were performed.

The effect of mutant htt on the differentiation capacity of HD-DPSCs was further determined by quantitative measurement on the expression of lineage specific markers including osteopontin (osteogenic marker), lipoprotein lipase (adipogenic marker) and collagen II (chondrogenic marker) by Q-PCR (Figure 3). There was no detectable expression of lipoprotein lipase and collagen II prior to induction. On the other hand, osteopontin was expressed before induction and was increased in some of the DPSC lines after induction. Variations among cell lines were also observed (Figure 3).

### Cell surface antigen profile

The expression profile of cell surface antigens is another method for determining the similarity between cell types and is often used to isolate ASCs. A total of 13 cell surface antigens were examined and compared to published reports. rHD/G-DPSCs were positive for CD29^+^, CD73^+^, CD90^+^, CD166^+^, CD59^+^, CD44^+ ^and CD105^+^, all of which are BMSC markers (Table 1). However, markers specific for hematopoietic cells, including CD14, CD34, and CD45, were not detected (Table 1). rHD/G-DPSCs were also negative for CD18, CD24 and CD150 (Table 1). These results were comparable to the expression profiles of the rG-DPSCs and other higher primates (Table 1) [2,4,23,24,27]

**Table 1:** Cell surface antigen profiles of ASCs derived from monkey, chimpanzee and human

	rHD/G-DPSCs	rDPSCs	hBMSCs	**ChDPSCs **[2]	**rBMSCs **[23]	**hDPSCs **[3]	**hBMSCs **[24]
CD14*	-	-	-	-	-	-	-

CD18	-	-	-	-	ND	ND	-

CD24	-	-	-	-	ND	ND	-

CD29	+	+	+	+	+	+	+

CD34*	-	-	-	-	-	-	-

CD44	+	+	+	+	ND	+	+

CD45*	-	-	-	-	-	-	-

CD59	+	+	+	+	ND	ND	+

CD73*	+	+	+	+	ND	ND	+

CD90*	+	+	+	+	+	ND	+

CD105*	+	+	+	+	ND	ND	+

CD150	-	-	-	-	ND	-	-

CD166	+	+	+	+	+	+	+

*Important markers for hBMSCs

ND-Not Determine

### Expression of mutant htt in rHD/G-DPSCs

**Figure 4:**
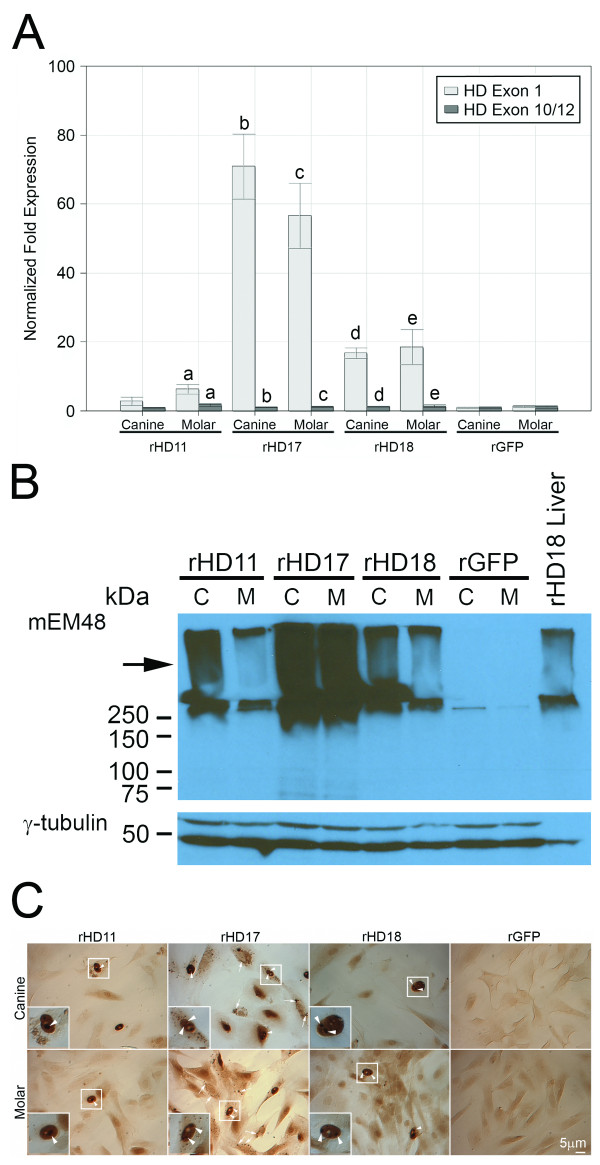
**Expression of the mutant htt**. (A) Quantitative analysis of htt transcripts in rHD/G-DPSCs and rG-DPSCs. qRT-PCR was used to measure the amount of htt transcripts. In addition to measuring the expression level of exon1, which is composed of both endogenous htt and the mutant htt, an additional set of primers measuring the expression level of Exon10/12 was used to show the level of normal htt alleles. The htt expression was significantly increased in all rHD-DPSC lines compared to rGFP. Column shares the same alphabet are significantly different (P < 0.05). (B) Western analysis demonstrated the accumulation of oligomeric htt (arrow) at high molecular weight (> 250 kD) that can be clearly seen in upper portion (Arrow) of a gradient polyacrylamide gel (upper panel). Antibody to γ-tubulin (bottom panel). (C) Subcellular distribution of mutant htt in rHD/G-DPSCs. DPSCs of rHD11, rHD17, rHD18 and rGFP were immunostained with mEM48. Transgenic mutant htt aggregates (arrows) and nuclear inclusions (arrowheads) were only observed in rHD11, rHD17 and rHD18 but not in DPSCs of rGFP. Scale bar = 5 μm.

In order to determine the effect of mutant htt on the properties of rHD/G-DPSCs, the expression of mutant htt and the formation of oligomeric htt were evaluated. Expression of mutant htt was significantly increased in all HD-DPSCs determined by Q-PCR (Figure 4A). Among HD-DPSC lines, rHD17 had the highest expression of mutant *htt*, while rHD11 has the lowest (Figure 4A). The formation of mutant htt aggregate was revealed by using western blotting analysis (Figure 4B) and immunostaining (Figure 4C) using mEM48, a monoclonal antibody that binds to human htt enhanced with a polyQ expansion. Nuclear inclusions and mutant htt aggregates were observed in all rHD/G-DPSCs, but not in rG-DPSCs (Figure 4C). Among the three HD monkeys, rHD17 had the most extensive intranuclear inclusions and cytoplasmic mutant htt aggregates compared to rHD11 and rDH18. Western blotting analysis further confirmed the presence of oligomeric htt at a high molecular weight (> 250 kD) in the upper portion of a gradient polyacrylamide gel (Figure 4C) in all HD-DPSC cell lines. Again, rHD17 had a more intense band than rHD11 and rHD18, suggesting the presence of more oligomeric htt. The observation of htt by immunostaining of rHD/G-DPSCs was consistent with the expression level of mutant htt determined by Q-PCR and the extent of oligomeric htt demonstrated by Western blot analysis.

## Discussion

Latest advancements in therapeutic applications of ASC have driven our interest in evaluating a patient's own DPSCs as an alternative source for cell therapy. Although recent studies have demonstrated the differentiation potential of DPSCs toward neuronal lineage [5,6], in-depth characterization such as biofunctions *in vivo *will further determine the therapeutic potential of DPSC derived neural cells. While therapeutic applications of DPSCs in cell replacement therapy are at preliminary stage, continued development in differentiation protocol and *in vivo *validation are important steps. Unlike cell replacement, endogenous stem cells are expected to divide and differentiate under the influence of the DPSC graft and the microenvironment [4,5,12].

In this study, HD-DPSCs were derived from teeth buds of miscarried HD monkeys and HD monkeys that died soon after birth. While the biofunctions of HD-DPSCs may vary from those derived from adult individuals, our findings suggested rHD/G-DPSCs retain properties comparable to ASCs [3,4,24,27]. Additionally, the impact of age on the biofunctions of DPSCs has not been fully addressed and it is important for determining future applications of DPSCs derived from individuals at different ages. Although we speculate the biofunctions of DPSCs will not be impacted significantly by age, the derivation efficiency of DPSCs may vary due to the number of stem cells existed in the dental pulps. While the present study is aimed to determine if DPSCs with inherited genetic defect retain ASC properties, our future goal is to determine if DPSCs derived from living HD monkeys or patients can be used to derive neuronal cell types for replacement therapy. Moreover, genetic correction of HD-DPSCs may be considered prior cell transplantation because HD-DPSCs may not have longevity and may not function in the same way as DPSCs derived from normal animals.

Multipotent differentiation capability was evaluated by *in vitro *differentiation into osteogenic, adipogenic and chondrogenic lineages. Differentiation into these three lineages is considered one of the basic requirements for BMSCs, which is widely applied in ASCs [24]. All rHD/G-DPSCs differentiated to osteogenic, adipogenic and chondrogenic lineages, which were comparable to rG-DPSCs and published reports (Figure 1) [2-4,27,28]. Recent studies have suggested that DPSCs are also capable of differentiating into neurons [5,6,8,29]. Although the current paradigm of ASC therapy is primarily based on the homing nature of ASCs [12,30,31] and their capability of eliciting a local repair response [4,12,30,32], their ability to differentiate into multiple cell types including neurons may be therapeutically relevant. While all tested cell lines were capable of differentiating into three different lineages, quantitative studies on specific lineage markers suggested variations in differentiation capacity among DPSC cell lines exists. Therefore, whether DPSCs derived from HD monkeys or normal rhesus monkeys have more preferred lineages upon differentiation cannot be concluded.

## Conclusions

Our results suggested that DPSCs derived from HD monkeys retain ASC properties, thus one may consider that these cells also retain biofunctions comparable to those derived from healthy individuals [4]. This study supports the potential future therapeutic application of DPSCs from patients with genetic disorders such as HD. We demonstrated that DPSCs from HD monkeys are comparable in all aspects that define ASCs. Thus DPSCs derived from patients with inherited genetics diseases hold great promise as an alternative source for personal cell therapy.

## Methods

All transgenic HD monkeys were housed under the guideline of the IACUC approved procedures and the support of the Division of Animal Resources at the Yerkes National Primate Research Center (YNPRC). All procedures were approved by YNPRC/Emory Animal Care and Biosafety Committees.

### Generation of transgenic monkeys [22]

Transgenic Huntington's monkeys were generated as described by Yang and colleagues [22]. In brief, lentiviruses carrying the Exon 1 of the htt gene containing expanded CAGs under the control of ubiquitin promoter were used to infect metaphase II arrested rhesus monkey oocytes followed by fertilization and embryo transfer into surrogate females.

### Isolation and culture of DPSCs [4,25]

The teeth germs/buds were recovered from monkeys miscarried at four months gestation (rHD11 and rGFP) and died soon after birth (rHD17 and rHD18). The teeth germs/buds were then digested in 3 mg/ml collagenase type I and 4 mg/ml dispase (Invitrogen, Inc) for one hour at 37°C. Single cell suspension was filtered through a 70 μm cell strainer and was then cultured in DPSC culture medium (α-MEM (Invitrogen, Inc) supplemented with 20% FBS (Atlanta Biologicals, Inc), 2 mM glutamine, 100 units/ml penicillin and 100 μg/ml streptomycin (Invitrogen, Inc.)) at 37°C with 5%CO_2_.

### Adipogenic, osteogenic and chondrogenic differentiation [2,4,25]

*For adipogenic differentiation*, cells were seeded at 400 cells/35 mm tissue culture dish and cultured for 11 days in DPSC medium. On day 11, the medium was supplemented with 5.0 μg/ml insulin, 50 μM indomethacin, 1 μM dexamethasone, and 0.5 μM IBMX, which was replaced every 3-4 days for a total of three weeks. The culture was then fixed in 4% paraformaldehyde (PFA) and stained with 0.0125% Oil-Red-O in isopropanol for 20 minutes at RT followed by a thorough wash and microscopic examination.

*For osteogenic differentiation*, cells were prepared as described for adipogenic differentiation until day 11. On day 11, the medium was supplemented with 1 nM dexamethasone, 50 uM L-Ascorbic acid 2-phosphate sesquimagnesium salt, 20 mM β-glycerolphosphate, and 50 ng/ml L-thyroxine sodium pentahydrate, and was replaced every 3-4 days for a total of three weeks. The culture was then fixed in 4% PFA and stained with 1% Alizarin Red S, pH 4.1 for 20 minutes at RT followed by a thorough wash and microscopic examination.

*For chondrogenic differentiation*, 2.5 × 10^5 ^DPSCs were centrifuged in a 15 ml conical tube at 1,000 rpm for five minutes. The pellet was maintained in a DPSC medium supplemented with ITS-plus premix (BD Biosciences) to a final concentration of 6.25 ug/ml insulin, 6.25 ug/ml transferrin, and 6.25 ng/ml selenious acid. Additionally, 5.35 ug/ml linoleic acid, 1.25 mg/ml bovine serum albumin, 50 μg/ml Ascorbate 2-phosphate, 40 μg/ml L-proline, 100 μg/ml Sodium pyruvate, 100 nM Dexamethasone, 100 units/ml penicillin, 100 μg/ml streptomycin and 10 ng/ml TGF-β3 (R&D Systems) were also supplemented. Medium was replaced every 3-4 days for a total of four weeks. The pellets were then fixed in 4% PFA overnight. The paraffin-embedded sections (4-5 μm) were stained with 1% Alcian blue in 10% sulphuric acid solution for 15 minutes followed by a thorough wash and microscopic examination.

### Quantitative Real-Time PCR (Q-PCR)

RNA from cell samples was prepared using RNeasy Mini kit (Qiagen). An equal amount of total RNA was used to synthesize cDNA followed by Q-PCR using iQ5 Real-Time PCR Detection System (Bio-Rad). Specific-qPCR primer sets targeting stem cell and differentiation markers were used (Additional file 1).

### Immunocytochemistry and fluorescent microscopy [22]

Cell samples were fixed using 4% PFA, permeabilized and blocked. The expression of mutant htt was then incubated with mEM48. The mEM48 (1:200) immunoreactive product was visualized with the avidin-biotin complex kit (Vector ABC Elite). For fluorescent microscopy, the samples were examined with an Olympus BX51 epifluorescent microscope.

### Western Blotting [22]

Total protein was extracted and concentrated for analysis using the Bradford Assay (Bio-Rad, Inc.). Equal amounts of the protein were boiled prior to polyacrylamide gel electrophoresis, the proteins were transferred onto a PVDF membrane (Millipore Immobion P, Millipore, Inc.) using Bio-Rad's transblot. The membrane was then blocked, incubated with primary antibody, secondary antibody, and detected using Amersham's ECL kit (Amersham, Inc.). The amount of protein was quantified using a densitometer.

### Flow cytometry analysis [2,4]

Cell samples at 2 × 10^5 ^cells/tube were stained with FITC or PE-conjugated anti-CD14, -CD45, -CD59, -CD73, -CD90, -CD150, -CD166, -IgG1k, -IgG2ak (BD Pharmingen), or anti-CD18, -CD24, -CD29, -CD34, -CD44 (BD Biosciences), or anti-CD105 (eBioscience). After incubating 20 minutes at RT in the dark, cells were washed with 2 mL FACS wash solution (dPBS+1%BSA+0.1%NaN_3_) and centrifuged five minutes at 230 × g. Supernatant was removed and cells were fixed with 1% formaldehyde. All data was acquired using a FACS Calibur (Becton Dickinson) and analyzed using CellQuest (Becton Dickinson) and Flowjo software (Treestar, Inc.).

### Statistical analysis

Data analyses were carried out using the *Student *t-test.

## Authors' contributions

BRS, PHC: DPSCs culture, in vitro differentiation, molecular analysis; JJY: DPSCs derivation and culture; SHY: Histological analysis; AHCH: DPSCs derivation and conceptual design; AWSC: DPSCs derivation, *in vitro *differentiation, conceptual design, preparation and approval of manuscript. All authors have read and approved the final manuscript.

## Supplementary Material

Additional file 1 **Supplemental Methods**. Primer sets for stemness and differentiation markers. Click here for file
