# Supplementary material for: Characterization of dental pulp stem/stromal cells of Huntington monkey tooth germs
Source: BMC Cell Biol. 2011 Sep 12;12:39. doi: 10.1186/1471-2121-12-39 (PMC3189880; doi:10.1186/1471-2121-12-39)
Supplement: Additional file 1 — Supplemental Methods. Primer sets for stemness and differentiation markers. [file 1471-2121-12-39-S1.DOCX]

**Supplemental Methods**

**Primer sets for stemness and differentiation markers:**

Stemness markers:

Oct 4 (Oct4-F: 5’-GCA ACC TGG AGA ATT TGT TCC T-3’ and Oct4-R: 5’-GGG CGA TGT GGC TGA TCT-3’)

Rex-1 (Rex1-F: 5’-GCC AAG ACG AGA CGC CAG AA-3’ and Rex1-R: 5’-CCC ACA GGC TCC ATT TCC GC-3’)

Nanog (Nanog-F: 5’-TGA AGC ATC CGA CTG TAA AGA ATC-3’ and Nanog-R: 5’-CAT CTC AGC AGA AGA CAT TTG CA-3’)

Differentiation markers:

Osteopontin (osteopontin-F: 5’-CAG TGA TTT GCT TTT GCC TCC T-3’ and osteopontin-R: 5’-GCT TTC GTT GGA CTT ACT TGG A-3’)

Lipoprotein lipase (LPL-F: 5’-AGC CAA AAG AAG CAG CAA GAT G-3’ and LPL-R: 5’-GAT GTT CTC ACT CTC AGC CAC-3’)

Collagen II (collagen II-F: 5’-GGA GAG CCT GGA GAT GAT GG-3’ and collagen II-R: 5’-CCC AGG CAG ACC GAC GAT G-3’)

Huntingtin gene: HD Exon1 (HD Exon 1-F: 5’-ATG GCG ACC CTG GAA AAG CT-3’and HD Exon1-R: 5’-TGC TGC TGG AAG GAC TTG AG-3’), HD Exon10/12:(HD Exon10-F: 5’-TCA AGA AAA CAA AAA GGC AAA GTG-3’ and HD Exon12-R: 5’-GTG GAA ACC CCT GAA GAA GCA-3’)

Internal control 18S(18S-F: 5’- CGG CTA CCA CAT CCA AGG AA -3’ and 18S-R: 5’- CCT GTA TTG TTA TTT TTC GTC ACT ACC T- 3’).
